# Pubic Osteomyelitis Identified on Follow-Up MRI Following Adductor Myositis in a Patient With Escherichia coli Bacteremia

**DOI:** 10.7759/cureus.84945

**Published:** 2025-05-28

**Authors:** Hisashi Ishikawa, Kazuki Ocho

**Affiliations:** 1 Department of Internal Medicine, Ishikawa Hospital, Toukoukai Medical Corporation, Tsuyama, JPN

**Keywords:** adductor myositis, escherichia coli bacteremia, follow-up mri, musculoskeletal infection, pubic osteomyelitis

## Abstract

A previously healthy 54-year-old man presented with a high-grade fever and bilateral groin pain. Initial non-contrast imaging failed to identify an infectious focus, while blood cultures revealed the presence of *Escherichia coli*. Pelvic MRI on hospital day 5 demonstrated bilateral adductor brevis myositis without osseous involvement. Despite antimicrobial therapy, symptoms persisted. Follow-up MRI on hospital day 13 revealed new pubic bone marrow edema, confirming a diagnosis of pubic osteomyelitis. The patient was successfully treated with a 12-week course of antimicrobial therapy, resulting in clinical resolution.

This case illustrates a key diagnostic challenge in gram-negative bacteremia: musculoskeletal infections can evolve dynamically, and early imaging may underestimate the extent of the disease. The anatomical continuity between muscle and bone enables the contiguous spread of infection. In this case, symptom-guided follow-up MRI was instrumental in detecting delayed bone involvement and optimizing treatment decisions. Clinicians should consider interval imaging when initial studies are inconclusive and symptoms persist.

## Introduction

Musculoskeletal infections secondary to gram-negative bacteremia are diagnostically challenging, particularly when no clear source of infection is identified on initial evaluation [1]. *Escherichia coli*, although commonly associated with urinary tract infections, can also cause metastatic infections, such as pyomyositis and osteomyelitis [2]. Among imaging modalities, MRI is considered the most sensitive for detecting soft tissue and marrow abnormalities in these conditions [3].

Pubic osteomyelitis is an uncommon but clinically significant form of bone infection, typically associated with trauma, pelvic procedures, or urologic interventions. Its presentation in immunocompetent adults without predisposing factors is particularly rare and may be misdiagnosed or diagnosed late due to its variable symptoms and low clinical suspicion [2]. The anatomical proximity of the pubic bone to the adductor muscle origins provides a plausible route for contiguous infection spread, especially in the setting of hematogenous seeding [4].

This report describes a case in which *Escherichia coli* bacteremia initially manifested as adductor myositis, later evolving into pubic osteomyelitis. A follow-up MRI, prompted by persistent symptoms, captured the progression. This case highlights the importance of clinical vigilance, symptom-guided reassessment, and the utility of interval imaging in the dynamic management of musculoskeletal infections [1].

## Case presentation

A 54-year-old man employed by the municipal water department presented with two days of high-grade fever (maximum 40°C), bilateral groin pain, and suprapubic pain. His occupation involved routine dam inspections with moderate physical exertion, but there had been no recent trauma or unusually intense activity. He had no history of diabetes mellitus, immunosuppression, or other significant comorbidities.

On admission (day 1), physical examination revealed tenderness over the suprapubic area and bilateral groins. Passive extension of the hips elicited pain, and ambulation was limited. Hip range of motion was moderately restricted due to pain, but there were no neurological deficits or joint effusions.

Initial laboratory investigations revealed a white blood cell count of 6730/μL, C-reactive protein (CRP) 17.2 mg/dL, and D-dimer 56.2 μg/mL. Urinalysis showed bacteriuria, although both initial and post-prostatic massage urine cultures were negative. The prostate was non-tender and non-enlarged on digital rectal examination. A summary of the patient’s laboratory findings on admission is provided in Table 1.

**Table 1:** Laboratory findings on admission WBC: white blood cells, Neut: neutrophil, Lym: lymphocyte, Mon: monocyte, Eos: eosinophil, Bas basophil, RBC: red blood cells, Hb: hemoglobin, Hct: hematocrit, MCV: mean corpuscular volume, MCH: mean corpuscular hemoglobin, MCHC: mean corpuscular hemoglobin concentration, Plt: platelets, PT: prothrombin time, APTT: activated partial thromboplastin time, D-dimer: fibrin degradation product, TP: total protein, Alb: albumin, T-Bil: total bilirubin, D-Bil: direct bilirubin, AST: aspartate aminotransferase, ALT: alanine aminotransferase, ALP: alkaline phosphatase, γ-GT: gamma-glutamyl transpeptidase, LDH: lactate dehydrogenase, CK: creatine kinase, T-CHO: total cholesterol, BUN: blood urea nitrogen, Cr: creatinine, UA: uric acid, Na: Sodium, K: potassium, Cl: chlorine, Ca: calcium, Glu: glucose, HbA1c: glycated hemoglobin, CRP: C-reactive protein, TSH: thyroid-stimulating hormone, NT-proBNP: N-terminal pro-brain natriuretic peptide, PSA: prostate-specific antigen, HBsAg: hepatitis B surface antigen, HCVAb: hepatitis C virus antibody Values are presented as absolute or percentage counts with reference ranges based on institutional standards.

Parameter	Result	Unit	Reference range
WBC	6730	/μL	3500-9000
Neut	94.0	%	42-74
Lym	3.9	%	18-50
Mon	1.8	%	2-9
Eos	0.0	%	0-7
Bas	0.3	%	0-2
RBC	539	×10^6^/μL	438-577
Hb	15.7	g/dL	13.6-18.3
Hct	47.4	%	40.4-51.9
MCV	87.9	fL	76-96
MCH	29.1	pg	27-32
MCHC	33.1	g/dL	31-51
Plt	9.9	×10^4^/μL	14.0-37.9
PT	12.9	_S_	8.0-12.0
APTT	29.8	_S_	26.0-38.0
D-dimer	56.2	μg/mL	0.0-1.0
TP	6.5	g/dL	6.5-8.2
Alb	4.0	g/dL	3.7-5.5
T-Bil	1.3	mg/dL	0.2-1.0
D-Bil	0.3	mg/dL	0.0-0.3
AST	25	U/L	10-40
ALT	22	U/L	5-45
ALP	49	U/L	38-113
γ-GT	23	U/L	0-79
LDH	184	U/L	120-245
CK	343	U/L	50-230
T-CHO	169	U/L	150-219
BUN	20.2	mg/dL	8.0-20.0
Cr	1.17	mg/dL	0.65-1.09
UA	4.1	mg/dL	3.6-7.0
Na	135.6	mmol/L	135-145
K	3.4	mmol/L	3.5-5.0
Cl	98.3	mmol/L	98-108
Ca	8.9	mg/dL	8.2-10.0
Glu	160	mg/dL	80-140
HbA1c	5.6	%	4.6-6.2
CRP	17.2	mg/dL	0.-0.29
TSH	0.786	mIU/mL	0.61-4.23
NT-proBNP	283	pg/mL	0-125
PSA	0.521	ng/mL	0.0-3.9
HBsAg	0.00	IU/mL	0.00-0.02
HCVAb	0.0	IU/mL	0.0-0.9
Urine-pH	5.5		5.0–8.0
Urine-protein	(3+)		(-)~(±)
Urine-RBC	10-19		0–4 /HPF
Urine-WBC	1-4		0–4 /HPF
Urine-nitrite	(-)		(-)
Urine-bacteria	(1+)		(-)

**Figure 1:**
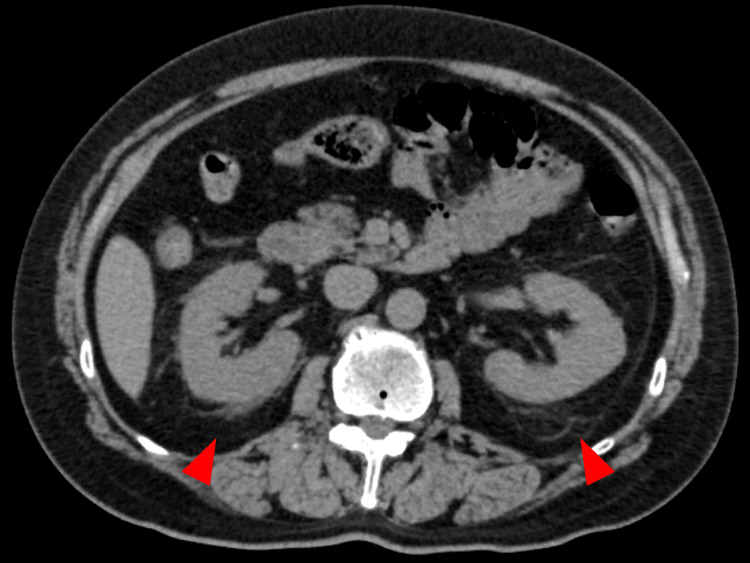
Non-contrast axial abdominal CT on admission Mild bilateral perinephric fat stranding is noted (arrowheads), raising suspicion for a urinary tract infection as the potential source of bacteremia. CT: computed tomography

Non-contrast abdominal CT performed on admission (day 1) demonstrated mild bilateral perinephric fat stranding (Figure 1). Empiric intravenous ceftriaxone (2 g/day) was initiated for suspected bacteremia of urinary or musculoskeletal origin. Blood cultures drawn on admission grew *Escherichia coli*, which was susceptible to β-lactam antibiotics and resistant only to azithromycin. Repeat blood cultures on day 17 were negative, confirming microbiological clearance.

**Figure 2:**
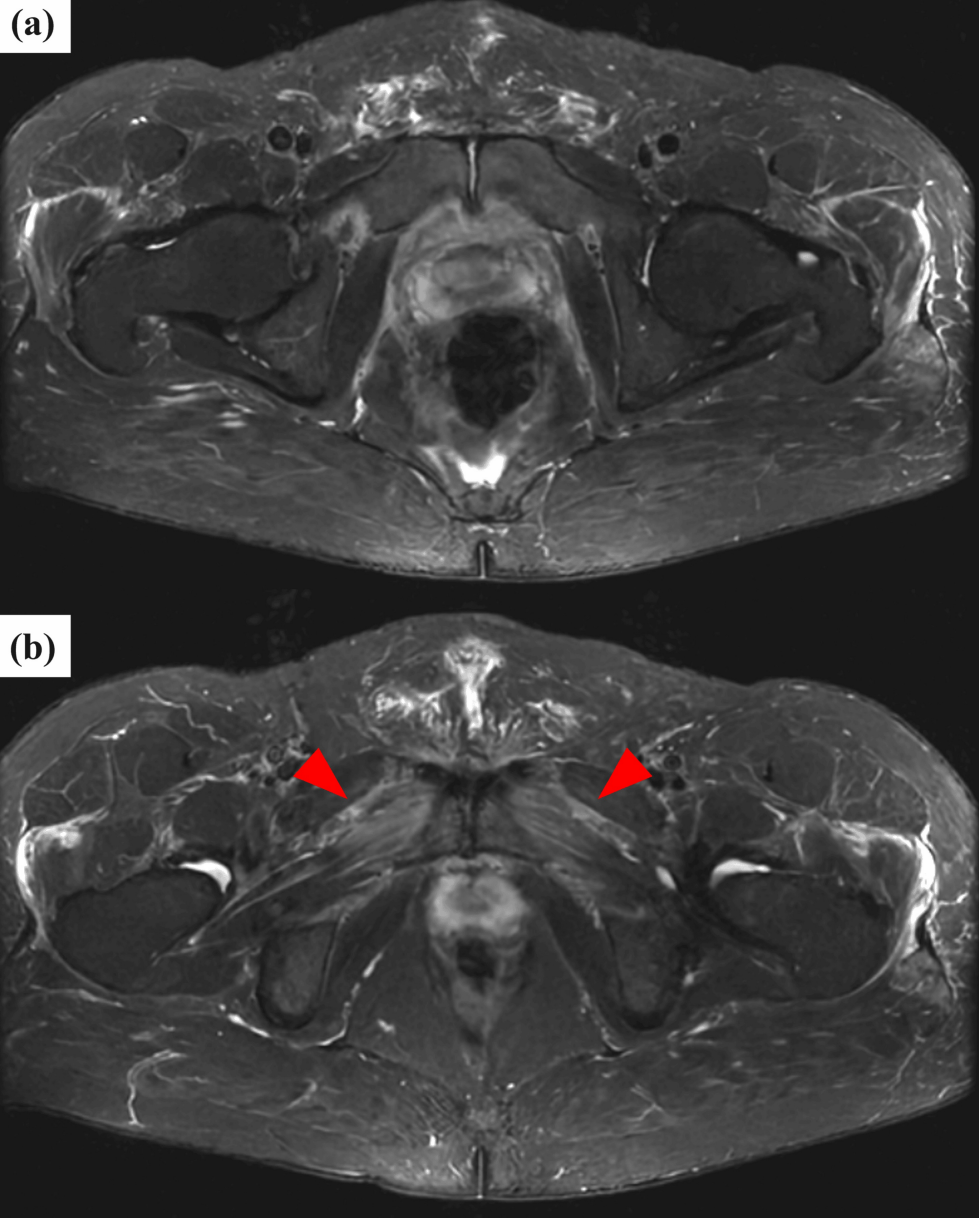
Pelvic MRI (STIR sequence) on hospital day 5 (a) No abnormal signal is observed in the pubic bone. (b) Symmetric high signal intensity is present in the bilateral adductor brevis muscles (arrowheads), localized to their proximal origin, consistent with early myositis and without osseous involvement at this stage. MRI: magnetic resonance imaging, STIR: short tau inversion recovery

**Figure 3:**
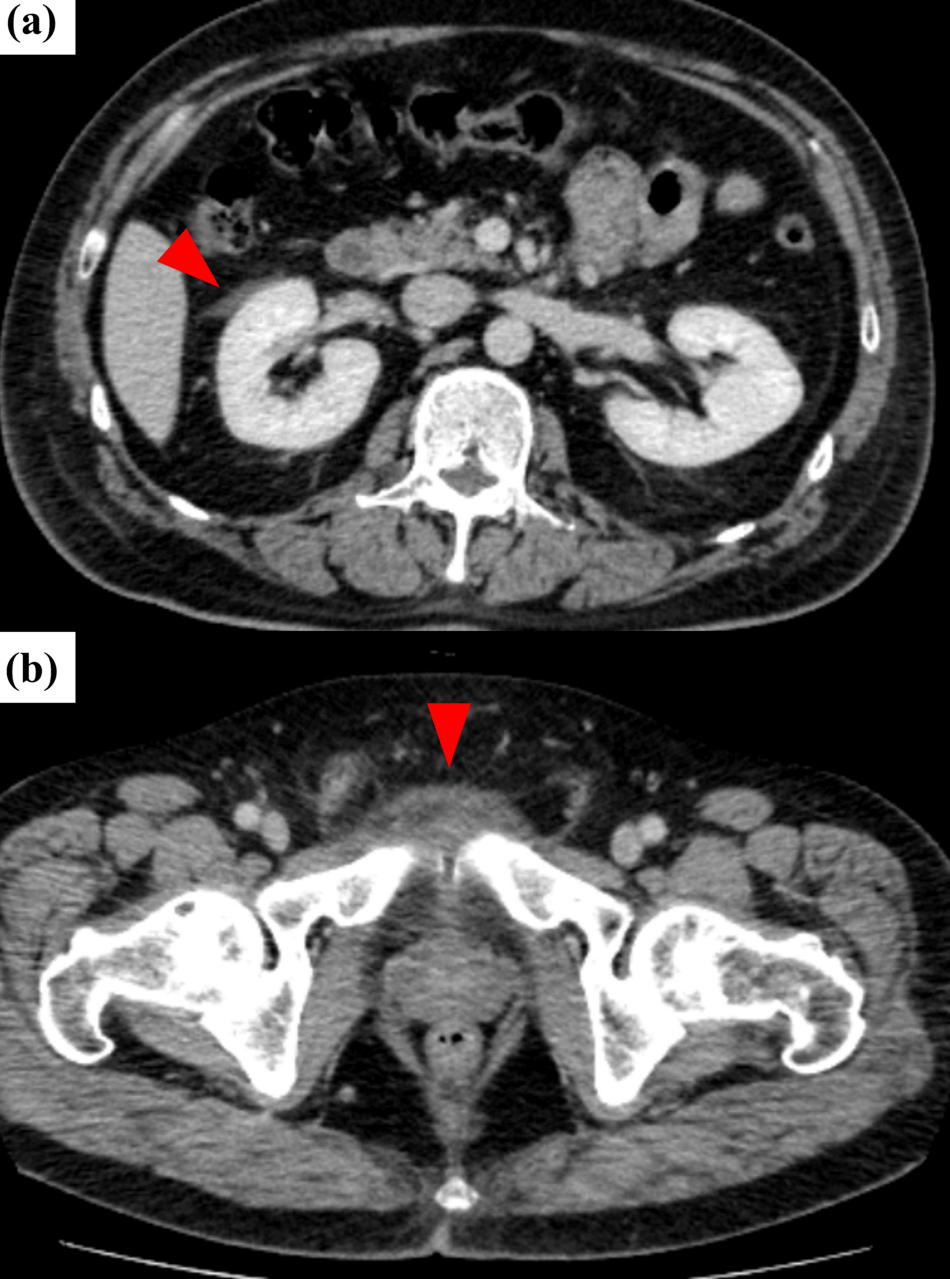
Contrast-enhanced abdominal and pelvic CT on hospital day 6 (a) A loculated fluid collection measuring approximately 2–3 cm is observed in the right perinephric space (arrow), without signs of abscess or gas formation, suggestive of transient inflammation. (b) Fat stranding anterior to the pubic symphysis, new compared to prior imaging, suggests evolving pelvic inflammation. CT: computed tomography

**Figure 4:**
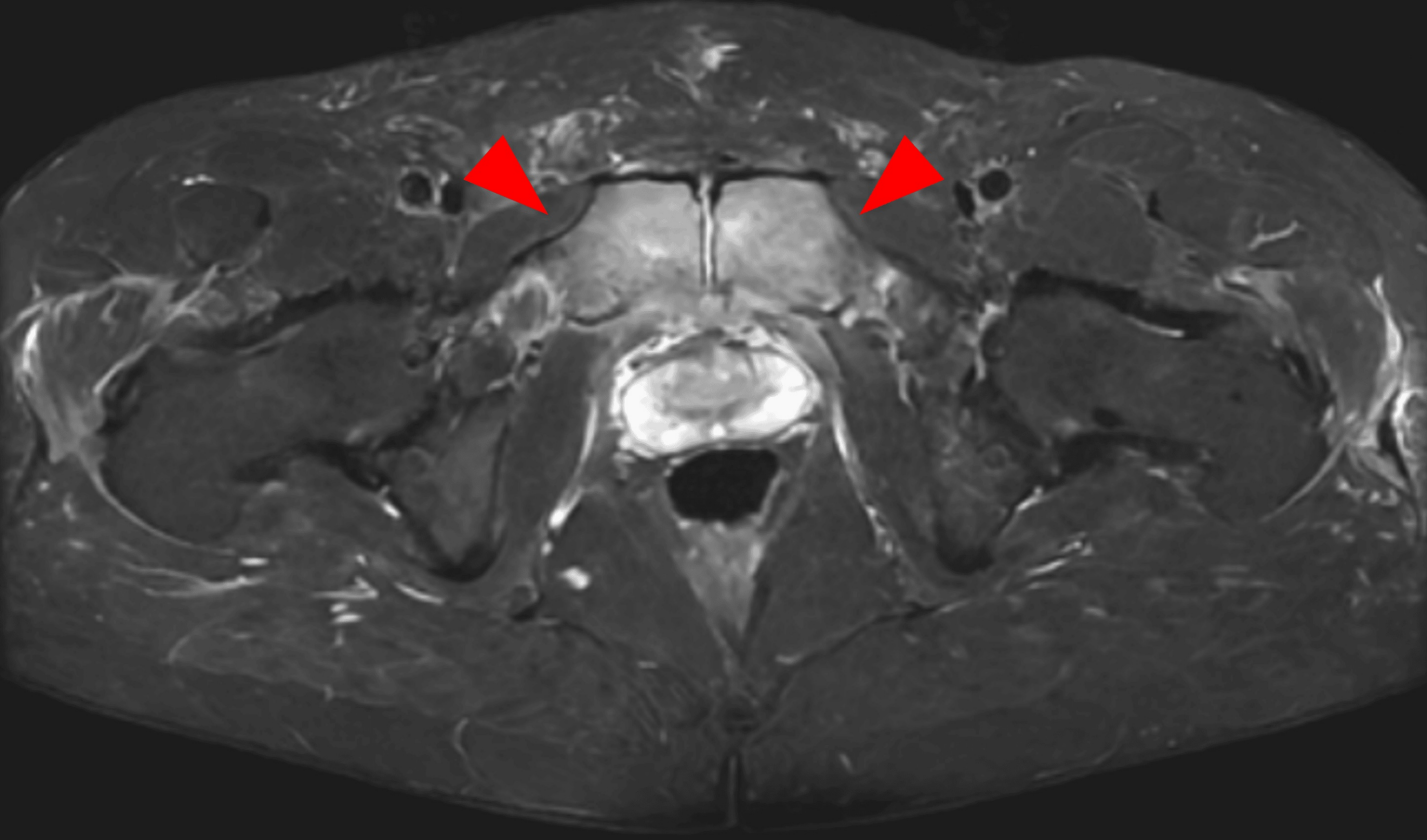
Pelvic MRI (STIR sequence) on hospital day 13 Diffuse high signal intensity involving the bilateral pubic bone marrow (arrowheads) is observed, consistent with osteomyelitis. This represents progression from earlier MRI findings of isolated myositis without osseous involvement. MRI: magnetic resonance imaging, STIR: short tau inversion recovery

On hospital day 5, pelvic MRI (STIR sequence) revealed bilateral adductor brevis myositis without osseous involvement (Figure 2). Symptoms persisted, and contrast-enhanced CT on day 6 showed perinephric fluid and fat stranding around the pubic symphysis (Figure 3). In response, antibiotics were escalated to sulbactam/ampicillin (3 g every six hours) from day 7 to day 13. Follow-up pelvic MRI on day 13 revealed new bone marrow edema in the pubic bone, consistent with osteomyelitis (Figure 4).

**Figure 5:**
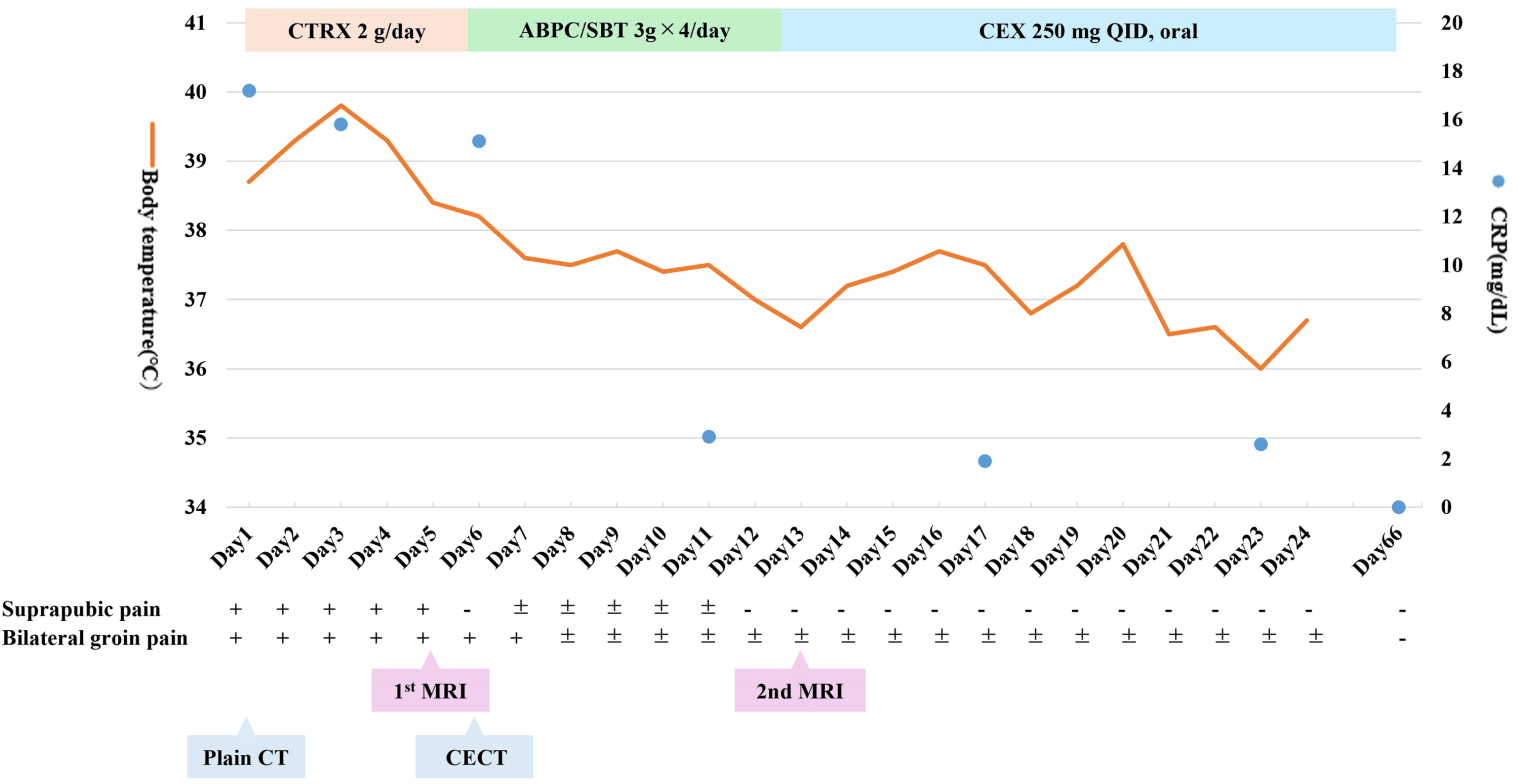
Clinical timeline integrating symptoms, body temperature, CRP, imaging, and antimicrobial therapy This figure summarizes the patient's clinical course, including body temperature (orange line) and CRP values (blue dots), over the course of hospitalization and follow-up. Antimicrobial regimens are shown as colored bars. Key imaging studies, including non-contrast CT, contrast-enhanced CT, and pelvic MRI, are annotated. The presence and improvement of suprapubic and bilateral groin pain are represented using symbols: “+” (present), “±” (improved), and “−” (resolved). CRP: C-reactive protein, CT: computed tomography, MRI: magnetic resonance imaging, CTRX: ceftriaxone, ABPC/SBT: ampicillin/sulbactam, CEX: cephalexin, QID: quater in die, CECT: contrast-enhanced computed tomography

The patient’s fever gradually resolved, and CRP levels declined during treatment. He was transitioned to oral cephalexin (250 mg QID) on day 14. He was discharged on day 23 in stable condition and completed a total of 12 weeks of antimicrobial therapy as an outpatient. At follow-up, he remained clinically well, without recurrence of symptoms. A timeline summarizing body temperature, CRP trends, antimicrobial regimen, imaging study dates, and the evolution of suprapubic and groin pain is presented in Figure 5.

## Discussion

This case illustrates the dynamic evolution of musculoskeletal infection in the context of *Escherichia coli* bacteremia, progressing from adductor myositis to pubic osteomyelitis. While *Escherichia coli* is a common cause of urinary tract infections, its ability to produce metastatic musculoskeletal involvement is less frequently appreciated, particularly in immunocompetent hosts [5]. The anatomical proximity of the adductor brevis origin to the pubic bone allows for direct contiguous spread of infection, as demonstrated radiologically and clinically in this case [2,4].

Initial MRI on hospital day 5 revealed bilateral adductor brevis myositis without osseous involvement, while follow-up MRI on day 13 confirmed new bone marrow edema consistent with osteomyelitis [6]. This progression underscores the importance of symptom-guided interval imaging, particularly when early studies are inconclusive or symptoms persist [6,7]. Although the utility of repeat imaging is well recognized in vertebral osteomyelitis, its role in non-vertebral cases is less frequently emphasized. Considering the anatomical variability and lower clinical suspicion in non-spinal sites, routine reassessment may be underutilized [7].

Empiric ceftriaxone therapy was selected for the initial management of suspected gram-negative bacteremia. This choice is supported by current clinical guidance and widely accepted as an appropriate first-line agent for community-acquired *Escherichia coli* infections, including those with potential musculoskeletal involvement [8]. Due to persistent symptoms and evolving imaging findings, therapy was escalated to sulbactam/ampicillin. Following clinical improvement and microbiological confirmation of β-lactam susceptibility, the patient was transitioned to oral cephalexin. This stepwise approach, combining initial intravenous therapy with a switch to oral agents based on clinical response and pathogen susceptibility, reflects principles supported by both observational studies and randomized trials evaluating antibiotic strategies for bone and joint infections [9]. Notably, the resistance profile of the isolate, susceptibility to β-lactams, and resistance to azithromycin were consistent with patterns commonly observed in community-acquired strains of *Escherichia coli* [10]. While molecular typing was not performed, the clinical pattern supports hematogenous seeding and contiguous spread.

This case also highlights the diagnostic challenge posed by pubic osteomyelitis, a rare and often underrecognized entity. Its presentation may mimic other causes of lower abdominal or pelvic pain, including athletic pubalgia, prostatitis, or pelvic insufficiency fractures, often leading to diagnostic delay. Awareness of the anatomical relationship between the pubic symphysis and surrounding musculature, and careful integration of imaging findings with evolving symptoms, is crucial for timely diagnosis.

Although the patient responded well to a 12-week course of antibiotics, the optimal duration of therapy for pubic osteomyelitis remains uncertain and should be individualized. Several guidelines and case series support durations ranging from six to 12 weeks, with decisions guided by clinical response, inflammatory markers, and radiologic improvement. In this case, the resolution of fever, normalization of CRP, and improvement on follow-up imaging collectively supported the completion of treatment.

## Conclusions

This case highlights a key diagnostic principle: musculoskeletal infections can evolve over time, and initial imaging may underestimate the extent of the disease. The progression from adductor myositis to pubic osteomyelitis illustrates the anatomical continuity that can facilitate the spread of infection, even in immunocompetent individuals.

Symptom-guided repeat MRI was crucial for identifying delayed osseous involvement. This emphasizes the importance of clinical judgment and interval imaging in patients with gram-negative bacteremia and musculoskeletal symptoms. A structured timeline helped visualize the interplay between symptoms, diagnostics, and therapy, offering a useful model for managing similar cases.
